# The Archbishop of Canterbury on Hospitals

**Published:** 1887-06-11

**Authors:** 


### The Archbishop of Canterbury on Hospitals.

Presiding at the first meeting of the Hospitals Fortnight, held at Lambeth Palace last Monday, the Archbishop of Canterbury said: I want to say with what pleasure I have placed this library at the services of the gentlemen who are organising this movement in Lambeth. It is, I consider, the most proper place in the world, though perhaps it is not very convenient in respect to its situation. We have to use it with the greatest care, because readers come from all parts of the world, and sometimes they are disturbed by meetings being held here; but I felt that Lambeth would be wanting to itself if we did not meet here on this occasion. I am glad that the resolution has taken the form of calling upon the clergy to do their very utmost for the hospitals. I believe that the clergy are always to be found at the service of these institutions, and it is very right, for they were of course the first healers. In my old church at Lincoln there is a structure which is surrounded by a series of deep niches which were the medicine niches of the Middle Ages. The founders of that cathedral felt that the care of the sick was a duty of the Church, and therefore they had this department reserved for them, and I daresay that in the fens of Lincolnshire there was a great deal of sickness. I took a very great interest in the erection of a hospital at Lincoln, indeed, I may say that really the magnificent building which has arisen is the child of that little square space in the cathedral with its medicine niches.

The Rev. Newman Hall has spoken of the way in which hospital work has sprung out of Christianity. I recently came across an interesting exemplification of this fact in the *Theodosian Codex*, which is divided very carefully into chapters and sections, and one of these chapters is headed: "Concerning the clergy, the places for the reception of strangers, and for the bringing up of orphans." Thus we see that in the Roman Empire the *xenodochia*, or houses for the reception of strangers, were so early formed. This publication, The Hospital, a number of which was kindly sent to me a short time ago, did open my eyes to the reasons why hospitals are so expensive. It would, I think, be an exceedingly bad policy if the Mansion House Committee were to do more than to satisfy themselves that the general management of hospitals was economical. We must put the hospitals, of course, into the hands of competent people, but year by year we must recognise the fact that the hospitals will grow more and more expensive. Some weeks ago in this paper there was a quotation made from *The Gentleman's Magazine* of a hundred years ago, with regard to the better ventilation and sanitation—as we should now say, for the word sanitation was not then in use—of hospitals, in which the writer—and it was only a hundred years back—pointed out in one of the London hospitals there was a hole over the doors of the wards, and he suggested that this mode of ventilation should be generally adopted! In Paris, the patients of the Maison Dieu were often to be found in their bed-dress standing upon the bridge over the Seine endeavouring to catch a little fresh air. The air of the hospital was so pestilential that they would rush down through the street and on to the bridge, and there stand gasping for breath. At the same time, at this great hospital every bed was occupied by several different patients during a day and a night. Now let us go into a hospital.

It does strike me, and it strikes most people, that if there is an institution, a place devoted to the purpose of the sick which is perfection, it is the hospital. When you see all the arrangements for nurses and attendants, the endless improvements for the treatment of patients, and the costliness of the appliances, it is no wonder that the hospital should increase in expensiveness. We ought to try to endeavour, therefore, to place still larger funds at the service of every one of our hospitals. When I say that a million people in London were last year directly benefited—served and treated as patients of the hospitals—I would ask how many people were indirectly benefited? Would it not be a very small compilation and below the mark if we said that there were three persons interested in the case of every person benefited. That gives us at once four millions—one million directly, and three millions indirectly, a number absolutely equal to the whole population of London.

A sum of £40,000 is wanted to meet the deficiencies of hospitals in London, and we know that that is not a great sum in London. When we think of a million people benefited, and the benefits conferred upon them, the amount is very small. Here in Lambeth we want £3,000, and I think we ought to raise it. If every single person present here to-day would interest others, the £3,000 would be raised at once. If you will carry these simple figures in your mind, and when you go from here will try to impress them upon others, I think the £3,000 can be raised.

Perhaps these few hasty remarks will not be inappropriately finished if I say that there is a still higher call upon us when the clergy are appealed to to raise funds. These funds are to be raised in places of worship, and therefore let us remember what is the real reason of our gathering together in places of worship. We gather there because we believe God hears our prayers. Whenever so small a sum is given, accompanied by earnest prayer, God will bless it. You know how in answer to prayer God has put money into the coffers of societies simply in answer to that prayer. When we go to carry our offerings into the treasury of God, let us therefore be very careful to accompany them with our prayers, that God will bless our offerings.

